# Supplementary material for: Comparative transcriptomics reveals desynchronisation of gene expression during the floral transition between Arabidopsis and Brassica rapa cultivars
Source: Quant Plant Biol. 2021 Apr 26;2:e4. doi: 10.1017/qpb.2021.6 (PMC10095958; doi:10.1017/qpb.2021.6)
Supplement: Supplementary file 1 [file S2632882821000060sup001.zip › S2632882821000060supp003.docx]

**Fig. S3:**

**Distribution of identified optimal registration function parameters.** A wide variety of different optimal parameters are identified for different genes, indicating that many differently synchronised processed occur, though some appear more common than others.
