# Supplementary material for: Comparative transcriptomics reveals desynchronisation of gene expression during the floral transition between Arabidopsis and Brassica rapa cultivars
Source: Quant Plant Biol. 2021 Apr 26;2:e4. doi: 10.1017/qpb.2021.6 (PMC10095958; doi:10.1017/qpb.2021.6)
Supplement: Supplementary file 1 [file S2632882821000060sup001.zip › S2632882821000060supp005.docx]

**Fig. S5:**

**Gene expression profiles of *FLC* paralogues in R-o-18,** expression of *FLC.A3a* (BRAA03G004170.3C) is dominant to the other FLC paralogues.
