# Supplementary material for: Comparative transcriptomics reveals desynchronisation of gene expression during the floral transition between Arabidopsis and Brassica rapa cultivars
Source: Quant Plant Biol. 2021 Apr 26;2:e4. doi: 10.1017/qpb.2021.6 (PMC10095958; doi:10.1017/qpb.2021.6)
Supplement: Supplementary file 1 [file S2632882821000060sup001.zip › S2632882821000060supp006.docx]

**Fig. S6:**

**Gene expression profiles of *FUL*, *FLC*, and *SOC1* in Arabidopsis and R-o-18**. Unlike in R-o-18, *FLC* expression declines before floral transition in Arabidopsis. In the Arabidopsis dataset, floral transition was determined to occur approximately 10d after germination (Klepikova *et al.*, 2015). In R-o-18, transition occurred by 17d. Expression is EdgeR normalised cpm. Brassica orthologues of Arabidopsis genes identified based on annotation in Chiifu v3 reference sequence (Zhang *et al.*, 2018).
