# Supplementary material for: Comparative transcriptomics reveals desynchronisation of gene expression during the floral transition between Arabidopsis and Brassica rapa cultivars
Source: Quant Plant Biol. 2021 Apr 26;2:e4. doi: 10.1017/qpb.2021.6 (PMC10095958; doi:10.1017/qpb.2021.6)
Supplement: Supplementary file 1 [file S2632882821000060sup001.zip › S2632882821000060supp007.pptx]

## Slide 1
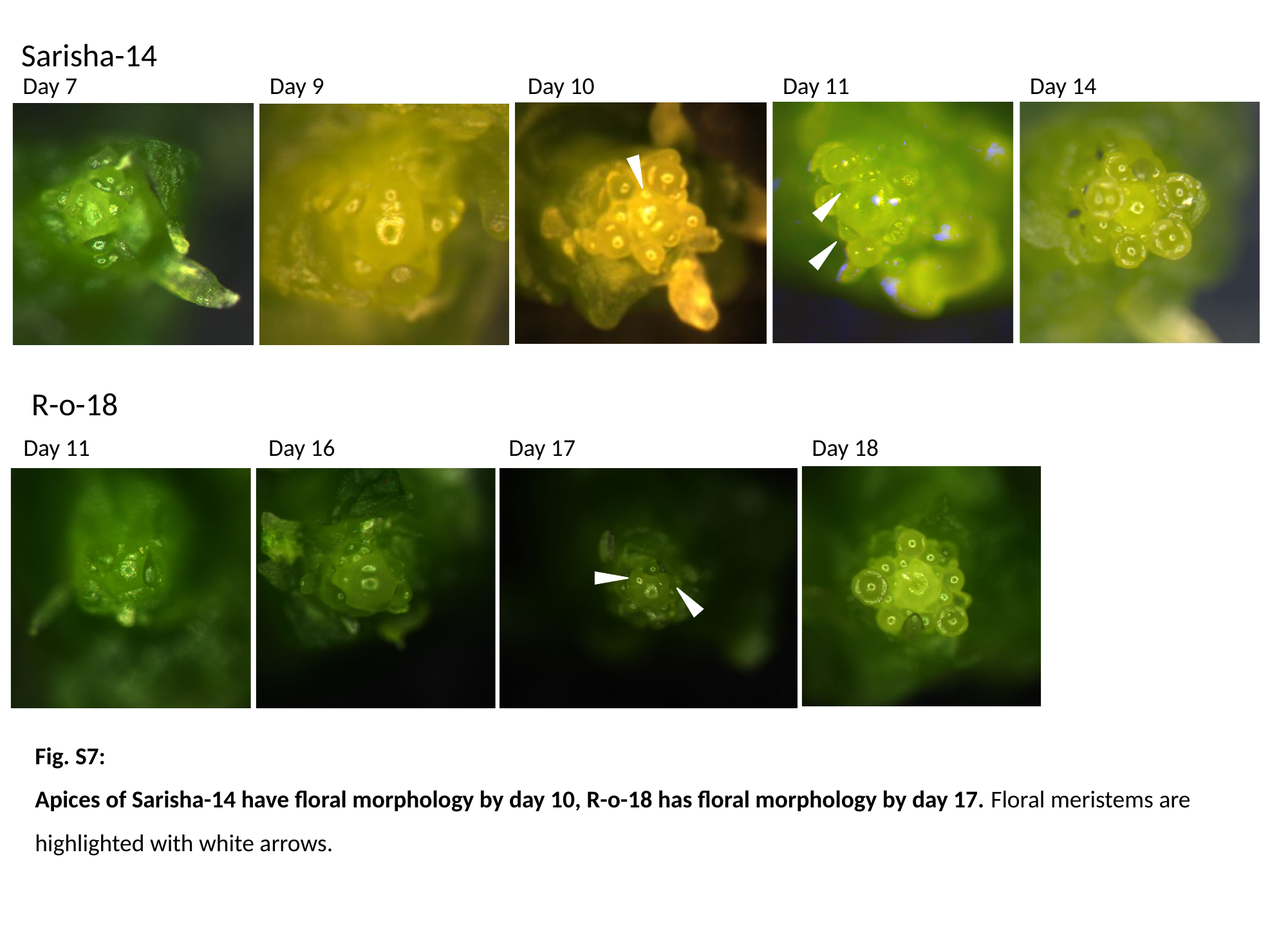

Sarisha-14
Day 7
Day 9
Day 10
Day 11
Day 14
R-o-18
Day 11
Day 16
Day 17
Day 18
Fig. S7:
Apices of Sarisha-14 have floral morphology by day 10, R-o-18 has floral morphology by day 17. Floral meristems are highlighted with white arrows.
